# On the tracks of an uninvited guest, the Asian tiger mosquito, *Aedes albopictus* in Cyprus

**DOI:** 10.1186/s13071-024-06651-5

**Published:** 2025-02-04

**Authors:** Riccardo Piccinno, Giulia Fiorenza, Marlen Ines Vasquez, Jeremy Bouyer, Gregoris Notarides, Ludvik Marcus Gomulski, Soteris Meletiou, Mustafa Akiner, Antonios Michaelakis, Federico Forneris, Giovanni Maga, Giuliano Gasperi, Anna Rodolfa Malacrida

**Affiliations:** 1 Department of Biology and Biotechnology, University of Pavia, Pavia, Italy; 2 Department of Chemical Engineering, Cyprus University of Technology, Limassol, Cyprus; 3 Insect Pest Control Subprogramme, Joint FAO/IAEA Centre of Nuclear Techniques in Food and Agriculture, International Atomic Energy Agency (IAEA), Vienna, Austria; 4 ASTRE, CIRAD, INRAE–University of Montpellier, Plateforme Technologique CYROI, Sainte-Clotilde, La Réunion France; 5 Recep Tayyip Erdogan University, Fener Rize, Turkey; 6 Laboratory of Insects & Parasites of Medical Importance, Benaki Phytopathological Institute, Athens, Greece; 7 Institute of Molecular Genetics of the National Research Council (IGM-CNR), Pavia, Italy

**Keywords:** *Aedes albopictus*, Asian tiger mosquito, Cyprus, Invasive, Vector

## Abstract

**Background:**

*Aedes albopictus*, the Asian tiger mosquito, which is listed among the world's 100 most dangerous invasive species, is the main vector of chikungunya, dengue and Zika viruses. This mosquito species has rapidly dispersed and invaded much of the globe assisted by its life history traits and high propagule pressure driven by human activities. *Aedes albopictus* is currently widespread across mainland Europe and the Mediterranean region, including the islands. Cyprus remained free of *Ae. albopictus* until October 2022, when specimens were recorded for the first time in Limassol district, including the port area. Understanding the processes associated with the introduction, expansion and establishment of this vector in Cyprus is of primary importance to mitigate its dispersal on the island, and to implement control methods to prevent disease outbreaks. A genetic analysis of these invasive specimens collected in Limassol district and in areas from the Central Mediterranean was performed to obtain a genetic portrait of the demographic history of the invasive mosquitoes on Cyprus.

**Methods:**

We applied highly polymorphic simple sequence repeat (SSR) markers to the *Ae. albopictus* mosquitoes collected in Cyprus and to specimens from Italy, France, Switzerland, the Balkans, Greece and Turkey to construct an SSR individual genotype dataset that would enable the invasion pattern of *Ae. albopictus* in Cyprus to be traced. Bayesian clustering analyses using STRUCTURE and BayesAss version 3 were employed to derive information on the degree of ancestry among Cypriot and Mediterranean mosquitoes and on recent mosquito movements both within Cyprus and between Cyprus and the Central Mediterranean areas.

**Results:**

The Cypriot mosquitoes appear to be highly polymorphic with no signs of genetic drift due to recent founder effects. An ongoing mosquito dispersal within the Limassol district was detected, suggesting the presence of established, hidden adventive populations. These mosquitoes share a high degree of ancestry with those in the Balkans and parts of northern Italy that border the Adriatic Sea.

**Conclusions:**

Considering the trade connections of Limassol port, Cyprus with the Balkans and the Adriatic Italian region, we hypothesise that these areas may be involved in the incursion of *Ae. albopictus* into Cyprus. As the Balkan and Italian mosquitoes display high competence for CHIKV, questions arise about possible arbovirus outbreaks in Cyprus and highlight the need to implement surveillance and control measures.

**Graphical Abstract:**

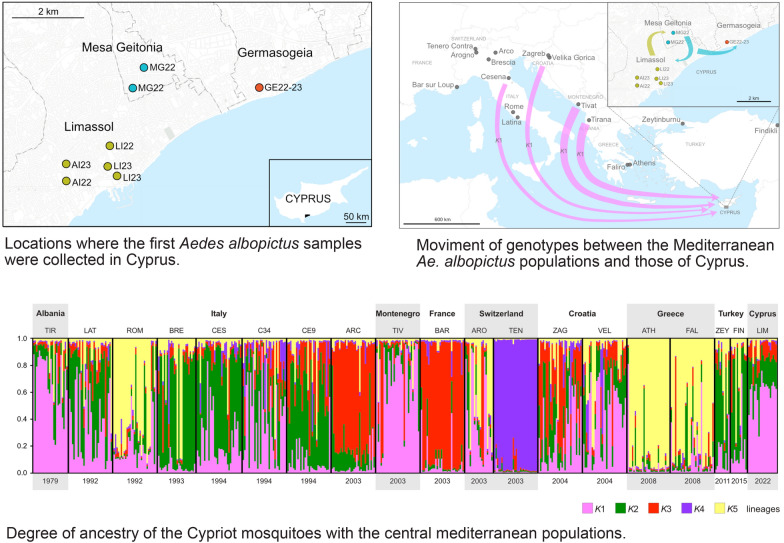

**Supplementary Information:**

The online version contains supplementary material available at 10.1186/s13071-024-06651-5.

## Background

Understanding the processes associated with the expansion and establishment of disease vector species into new areas is of primary importance in order to be able to interpret the causes of and predict and prevent disease outbreaks. This is especially true for *Aedes albopictus*, the Asian tiger mosquito, an arbovirus vector that has rapidly and successfully invaded much of the globe assisted by its life history traits and high propagule pressure driven by human activities [1–7]. From tropical Southeast Asia, where it was originally a zoophilic forest species [8], *Ae. albopictus* spread to the Indian and Pacific Ocean islands [9] and, beginning in the 1980s, rapidly extended its range across temperate regions in Europe, the Americas and Africa [10–12]. During its global colonisation process, admixture events among unrelated genomes played a major role in shaping the genetic makeup of the globally distributed adventive populations, creating genetic discontinuity and impacting their demographic histories [2, 3]. The Asian tiger mosquito is listed among the top 100 most dangerous invasive species [13]. It is the main vector of the chikungunya (CHIKV) and, to a lesser extent, dengue (DENV) and Zika (ZIKV) viruses. Moreover, experimental infections have shown the competence of this mosquito species for at least 20 arboviruses [14–17]. In countries where it has become established, it has been involved in local autochthonous transmission of chikungunya and dengue, including La Réunion, continental Europe, Africa, the Americas and Japan [18, 19].

During the invasion process, the dynamics of different mosquito genetic backgrounds have impacted arbovirus competence in the adventive populations, even at a fine spatial scale, so that geographically related populations may display differences in arbovirus competence [3, 20–23]. For example, in the Mediterranean area, colonisation occurred through various admixture events over time [2, 21, 24]. The presence of *Ae. albopictus* in Europe dates back to 1979 in Albania, where it was likely introduced from China, one of Albania’s few trading partners at the time [25]. It arrived in Italy in 1990 [26]. Since then, *Ae. albopictus* has spread to more than 25 European countries [27], including the southwestern islands of the Mediterranean [28]. The increasing presence of this mosquito, coupled with the rise in imported arbovirus cases [29–31], has led to local transmission of DENV and CHIKV in Croatia, France and Italy [32–37].

In response to the threat posed by invasive *Aedes* mosquitoes, many countries have implemented surveillance and control measures [10]. Among these is the Republic of Cyprus, which remained free of *Ae. albopictus* until July 2022 [38]. This island maintains significant trading networks with various European countries, including the UK, Germany, and Mediterranean countries, and supports a robust tourism industry [39]. Consequently, the risk of introducing *Ae. albopictus* into Cyprus has been a major concern [38, 40].

A horizon scanning study conducted in 2018–2019 [40, 41] using an expert-elicitation approach [42] produced a priority list of invasive alien species (IAS) that could threaten human health and the economy of Cyprus. *Aedes albopictus* and *Aedes aegypti* were included among the top 10 species, with high potential for introduction via aeroplanes, ships and vehicles and as contaminants on plants. By September 2019, an island-wide surveillance programme had been initiated. No *Ae. albopictus* mosquitoes were recorded until October 2022, when they were detected in the port area of Limassol municipality [28, 38]. The presence of these mosquitoes was also recorded in two other municipalities within the Limassol district: Mesa Geitonia and Germasogeia.

Emergency Action Plans were immediately developed to prevent the spread of *Ae. albopictus* and to implement suppression or eradication programmes, such as the sterile insect technique [38, 43]. A crucial aspect of these actions is to identify the plausible routes of introduction of this mosquito into Cyprus. Understanding these routes is essential for mitigating the spread and reducing the risk of outbreaks.

Using a genetic approach on some of the *Ae. albopictus* specimens recovered in 2022 in the Limassol district of Cyprus, we aimed to reconstruct their demographic history and infer the possible entry points into the island.

## Methods

### Mosquito samples

**Fig. 1:**
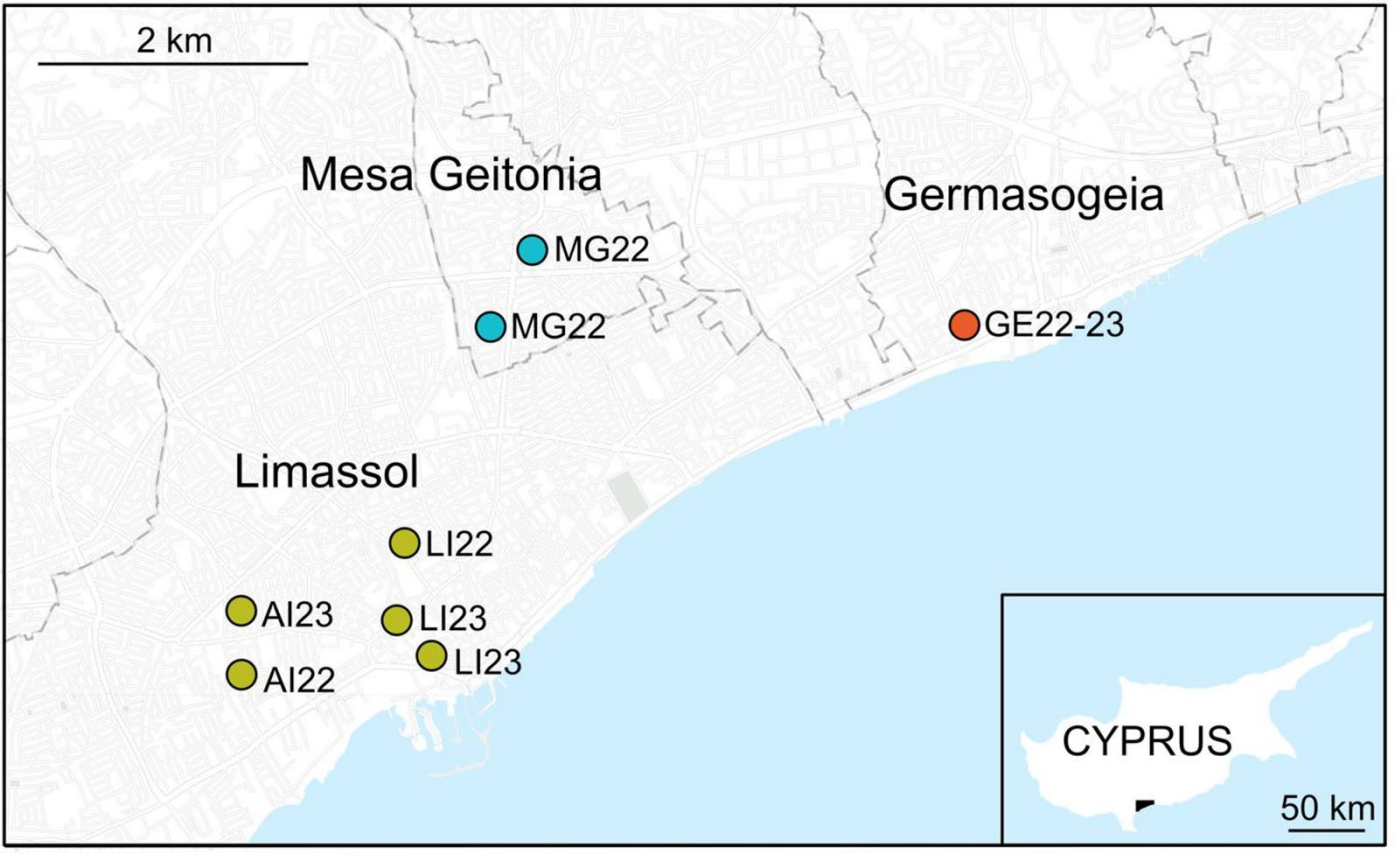
Geographical locations of the *Aedes albopictus* collection sites in Limassol district, Cyprus

Thirteen *Ae. albopictus* specimens from among the 69 retrieved by researchers of the Cyprus University of Technology for the first time in the Limassol district of Cyprus between October and November 2022 were sent to the University of Pavia as ethanol-preserved adults. These mosquitoes had been collected using BG-Sentinel™ traps (BGS; BioGents AG, Regensburg, Germany) and human landing catches (HLCs) in three municipalities of Cyprus: Germasogeia, Mesa Geitonia and Limassol (including two urban sites: Agios Ioannis and the Limassol port). Additionally, seven mosquitoes were collected in April 2023 in the Cyprus municipalities of Germasogeia and Limassol (both in Agios Ioannis and Limassol port sites; Table 1; Fig. 1). These specimens were morphologically identified as Asian tiger mosquitoes, *Ae. albopictus*, using two identification keys [44, 45].

**Table 1:** *Aedes albopictus* specimens collected in Limassol district, Cyprus October to November 2022 and April 2023

Sample ID	Municipality	Location	Collection date (day/month/year)	Sample code	Latitude	Longitude	Sex
LEMIT001	Limassol	Agios Ioannis	20/04/2023	AI23	34°40′33″ N	33°01′21″ E	M
LEMIT002	Limassol	Agios Ioannis	20/04/2023	AI23	34°40′33″ N	33°01′21″ E	F
LEMIT003	Limassol	Agios Ioannis	20/04/2023	AI23	34°40′33″ N	33°01′21″ E	F
LEMIT004	Limassol	Limassol port	20/04/2023	LI23	34°40′30″ N	33°02′09″ E	F
LEMIT005	Limassol	Limassol port	20/04/2023	LI23	34°40′23″ N	33°02′16″ E	F
LEMIT006	Limassol	Limassol port	20/04/2023	LI23	34°40′23″ N	33°02′16″ E	F
LEMIT007	Limassol	Agios Ioannis	29/10/2022	AI22	34°40′18″ N	33°01′21″ E	M
LEMIT008	Limassol	Agios Ioannis	29/10/2022	AI22	34°40′18″ N	33°01′21″ E	F
LEMIT009	Limassol	Agios Ioannis	29/10/2022	AI22	34°40′18″ N	33°01′21″ E	M
LEMIT010	Limassol	Limassol port	03/10/2022	LI22	34°40′49″ N	33°02′11″ E	F
LEMIT011	Limassol	Limassol port	03/10/2022	LI22	34°40′49″ N	33°02′11″ E	F
GEAIT001	Germasogeia	Germasogeia	20/04/2023	GE23	34°41′43″ N	33°04′55″ E	M
GEAIT002	Germasogeia	Germasogeia	29/10/2022	GE22	34°41′43″ N	33°04′55″ E	F
GEAIT003	Germasogeia	Germasogeia	29/10/2022	GE22	34°41′43″ N	33°04′55″ E	M
GEAIT004	Germasogeia	Germasogeia	29/10/2022	GE22	34°41′43″ N	33°04′55″ E	M
ΜGΑIT001	Mesa Geitonia	Mesa Geitonia	10/11/2022	MG22	34°41′43″ N	33°02′35″ E	Μ
ΜGΑIT002	Mesa Geitonia	Mesa Geitonia	10/11/2022	MG22	34°41′43″ N	33°02′35″ E	F
ΜGΑIT003	Mesa Geitonia	Mesa Geitonia	03/10/2022	MG22	34°42′02″ N	33°02′47″ E	F
ΜGΑIT004	Mesa Geitonia	Mesa Geitonia	03/10/2022	MG22	34°42′02″ N	33°02′47″ E	F
ΜGΑIT005	Mesa Geitonia	Mesa Geitonia	03/10/2022	MG22	34°42′02″ N	33°02′47″ E	M

*F* Female,* M* male

To obtain a genetic portrait of the demographic history of these Cypriot invasive mosquitoes, we included *Ae. albopictus* samples from 18 Central Mediterranean populations: eight from Italy, one from France, two from Switzerland, three from the Balkan area (Albania, Montenegro, Croatia), two from Greece and two from Turkey (Table 2). We chose these populations considering the progressive chronological invasion of *Ae. albopictus* in these countries. These samples were previously processed and analysed by our laboratory in terms of simple sequence repeat (SSR) allele frequencies and variability. The same SSR profiles were integrated into the Cyprus invasion analyses. Notably, as shown in Table 2, the Central Mediterranean populations were characterised for their competence for CHIKV, DENV and ZIKV [3, 21].

**Table 2:** Central Mediterranean populations of *Aedes albopictus* sampled in areas where the presence of this mosquito has been historically recognised

Country	Population	Code	First record^a^	Collection date (year)	*N*^b^	Latitude	Longitude
Albania	Tirana^c^	TIR	1979 [46]	2011	24	41°19′48″ N	19°49′48″ E
Italy	Brescia^c^	BRE	1993 [47]	2010	26	45°32′24″ N	10°13′12″ E
	Cesena^c^	CES	1994 [48]	2010	31	44°08′24″ N	12°15′00" E
	Cesena3 + 4^d,e^	C34	1994 [48]	2017	30	44°06′51" N	12°16′12" E
	Cesena9	CE9	1994 [48]	2017	30	44°10′05" N	12°17′44" E
	Latina	LAT	1992 [49]	2018	30	41°27′40" N	12°54′30" E
	Roma	ROM	1992 [49]	2017	30	41°54′00" N	12°29′00" E
	Arco	ARC	2003 [50]	2012	30	45°55′42" N	10°56′02" E
Montenegro	Tivat^d^	TIV	2003 [51]	2017	30	42°24′20" N	18°39′11" E
Switzerland	Tenero Contra^d^	TEN	2003 [52]	2017	30	46°10′27″ N	08°51′21″ E
	Arogno^d^	ARO	2003 [52]	2017	19	45°55′00" N	08°59′00" E
France	Bar sur Loup^c^	BAR	2003 [53]	2013	30	43°42′00″ N	06°59′24″ E
Croatia	Velika Gorica^d^	VEL	2004 [54]	2017	30	45°42′26" N	16°05′07" E
	Zagreb^d^	ZAG	2004 [54]	2017	30	45°50′09" N	15°58′40" E
Greece	Faliro^d^	FAL	2008 [55]	2017	30	37°55′51" N	20°41′58″ E
	Athens^c^	ATH	2008 [55]	2011	29	37°58′48″ N	23°43′48″ E
Turkey	Zeytinburnu	ZEY	2011 [56]	2021	10	40°59′23″ N	28°53′44″ E
	Findikli	FIN	2015 [57]	2023	11	41°16′15″ N	41°08′56″ E

^a^First record dates refer to the first observational record in the country where the sampling site is located

^b^Sample size used for microsatellite analyses

^c^Population samples evaluated for vector competence for chikungunya virus

^d^Population samples evaluated for vector competence for chikungunya, dengue and Zika viruses

^e^Sample comprises two simultaneous collections of mosquitoes sampled in two neighbouring sites within Cesena

### Microsatellite Analyses

We used SSRs previously validated as highly polymorphic markers to provide continuity with our published *Ae. albopictus* population data and also because their use allowed accurate biogeographic data analyses [2, 3, 58]. Eleven SSR loci (Aealbmic1, -2, -3, -5, -6, -9, -11, -14, -15, -16 and -17) were applied to the 20 mosquitoes collected in Cyprus to produce an SSR individual profiling dataset, with no major bias shown in the statistical analyses. These SSR loci were chosen for their high polymorphism; distribution across the genome, namely they map to different scaffolds of the *Ae. albopictus* Foshan and Rimini Genomes (GenBank identifiers GCA_001444175.2, GCA_035046485.1, and GCA_001574995.1) [59, 60] (our unpublished data); and proven efficiency as markers for detecting variability, even in relatively small samples [1]. Moreover, these loci have been used earlier to develop the individual genotype dataset for the Central Mediterranean populations considered here. The generated SSR genotype dataset was used to trace the invasion pattern of *Ae. albopictus* in Cyprus.

Genomic DNA was extracted from each mosquito collected in Cyprus using a standard method [61], and the quantity and quality of the DNA were evaluated using a Nanodrop ND-1000 spectrophotometer (Nanodrop Technologies Inc., Thermo Fisher Scientific, Waltham, MA, USA). PCR amplification and SSR fragment identification were performed as previously described [1]. To account for genotyping errors, automated binning of allele lengths was performed with TANDEM v1.09 [62], followed by manual checking.

### Genetic variability estimation

Variation within the Cypriot samples was estimated in terms of the average number of alleles (*N*_a_), effective alleles (*N*_e_), observed heterozygosity (*H*_o_), expected heterozygosity (*H*_e_), *F*-statistic and pairwise fixation index (*F*_ST_) using GenAlEx 6.5 [63]. The average number of alleles was also computed at the individual level (*N*_a_/*N*). The statistical significance of each *F*_ST_ value was assessed by comparing the estimated value with the value obtained using 10,000 matrix permutations and Bonferroni corrections. Principal coordinate analysis (PCoA) was performed based on *F*_ST_ values using GenAlEx 6.5 and visualised in a plot generated using ggplot2 [64] in R version 4.2.3 [65]. Molecular variance (analysis of molecular variance [AMOVA]) was estimated using the adegenet 2.1.10 [66] and poppr 2.9.4 [67] R packages.

### Population structure

Bayesian clustering analysis using STRUCTURE v2.3.4 [68] was performed to derive information on the degree of ancestry shared among the adventive mosquitoes in Cyprus and with the Mediterranean mosquitoes. Considering that STRUCTURE is sensitive to uneven sample size [69], for these analyses the SSR individual profiles of mosquitoes collected in Germasogeia, Mesa Geitonia and Limassol were merged and considered to be representative of Limassol district. The admixture model was employed, assuming independent allele frequencies. The analysis involved a burn-in phase of 500,000 iterations, followed by 1,000,000 Markov Chain Monte Carlo (MCMC) replications. For each potential number of clusters (*K*), we conducted 20 independent runs. The range of clusters (*K*) considered spanned from 1 to 19, representing the number of samples under study. To ascertain the optimal number of clusters (*K*), we employed STRUCTURE HARVESTER [70].

The appropriate number of genetic clusters was determined by plotting the log probability (L(*K*)) and Δ*K* across multiple runs, as implemented in STRUCTURE HARVESTER. The Greedy algorithm in CLUMPP v1.1.2 [71] was used to merge the independent runs, and the graphical representation of the co-ancestry percentages obtained was plotted using DISTRUCT v1.1 [72].

### Migration and population assignment

BayesAss v3.0.5.6 [73, 74] was employed to infer recent mosquito immigration among the three localities in Cyprus and between them and the 18 Central Mediterranean areas considered in the study. Parameters were tuned according to the programme manual's suggestions [75]: m = 0.3 (mixing parameter for migration rates), a = 0.7 (mixing parameter for allele frequencies) and f = 0.7 (mixing parameter for inbreeding coefficients). The final values were estimated through a MCMC of 30 million iterations with a burn-in of 10 million iterations. Convergence of the run was assessed by inspecting the MCMC trace using Tracer v1.7.2 [76].

## Results

### Variability and genetic diversity within and between the adventive mosquitoes in Limassol District

The 11 microsatellite loci, scored in 20 mosquitoes detected in 2022 and 2023 across Limassol, Mesa Geitonia and Germasogeia municipalities, showed a mean polymorphic information content (PIC) of 0.67. This value suggests that these loci are sufficiently informative to derive information on the genetic status of these mosquitoes and to infer their demographic history.

The mosquitoes from these municipalities display a high degree of variability, with the number of alleles per individual ranging from 0.42 in Mesa Geitonia to 0.55 in Germasogeia. The expected heterozygosity (*H*_e_) estimates for the samples collected in 2022 range from 0.36 in Limassol to 0.24 in Germasogeia (Additional file 1: Table S1). In Limassol, the variability was found to increase from October 2022 to April 2023.

**Fig. 2:**
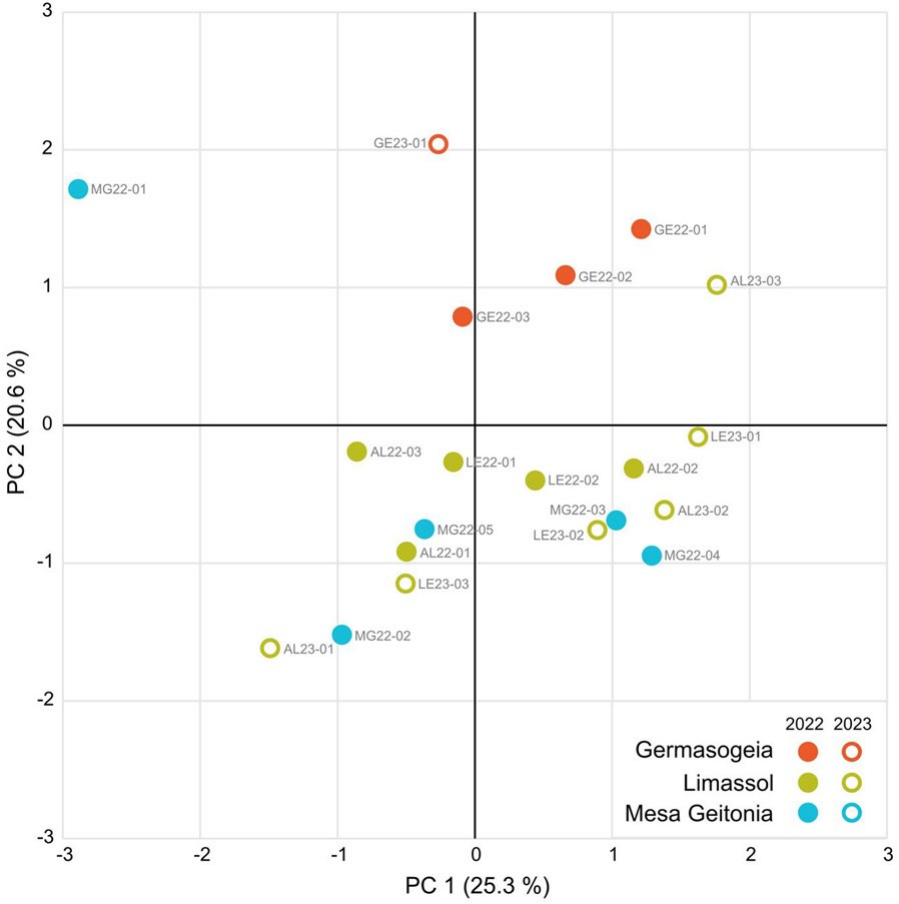
Principal coordinate analysis illustrating the relationships between the Cypriot *Aedes albopictus* samples collected in 2022 and 2023, respectively. PC, Principal coordinate

The PCoA plot in Fig. 2 highlights a certain degree of genetic heterogeneity among the mosquitoes collected in the three municipalities. While the mosquitoes collected in 2022 and 2023 from Limassol (Agios Ioannis and Limassol port) and Mesa Geitonia municipalities are mixed along the first axis (25.3%), those from Germasogeia are separated and spread along the second axis, which represents 20.6% of the total variation.

AMOVA analyses (Table 3) confirmed that most of the variance is present within individuals across the three municipalities (78%, row 1). However, it also revealed that the highest level of variance occurs when comparing mosquitoes from Germasogeia against those from Limassol and Mesa Geitonia combined (14.74%, row 3; Table 3). On the other hand, when Mesa Geitonia was compared against Limassol (variance = − 2.66%, row 4; Table 3), the results are not significant, and the variance is negative, indicating an absence of genetic structure.

**Table 3:** Analysis of molecular variance with different combinations of sampled *Aedes albopictus* from different municipalities in Cyprus

Comparison	Between municipalities				Between individuals within municipalities				Within municipalities			
	Variance	% var^a^	ϕ^b^	*P*_(adj)_^c^	Variance	% var	ϕ	*P*_(adj)_	Variance	% var	ϕ	*P*_(adj)_
Locations: Agios Ioannis, Limassol port, Mesa Geitonia, Germasogeia	0.25	5.26	0.05	0.04	0.80	16.74	0.18	0.01	3.71	78.00	0.22	0.00
Municipalities: Limassol, Mesa Geitonia, Germasogeia	0.38	7.78	0.08	0.01	0.76	15.62	0.17	0.02	3.71	76.60	0.23	0.00
Germasogeia vs (Limassol and Mesa Geitonia)	0.77	14.74	0.15	0.00	0.73	14.08	0.17	0.01	3.71	71.19	0.29	0.00
Limassol vs Mesa Geitonia	− 0.12	− 2.66	− 0.03	0.78	0.62	13.30	0.13	0.10	4.18	89.36	0.11	0.10

^a^The percentage of variance explained by each stratification

^b^Degree of differentiation

^c^*P*-value for the stratification performing 9999 permutations, adjusted using false discovery rate correction

### Inference of recent migration rates within the Limassol district

BayesAss 3 data on possible recent migrations among Limassol (including Limassol Port and Agios Ioannis samples), Germasogeia and Mesa Geitonia provide a general picture of mosquito movements among these three municipalities for 2022 (Table 4). The fraction of migrants (M) from Limassol to Mesa Geitonia and vice versa is fairly high (0.10 and 0.12, respectively). Lower M values have been estimated for migrations from these two municipalities to Germasogeia (0.08 and 0.09, respectively). In turn, Germasogeia was found to provide a lower fraction of emigrants to Limassol and Mesa Geitonia (0.07 and 0.07, respectively).

**Table 4:** Migration rates estimated between the samples of *Aedes albopictus* collected in 2022 and 2023 in the municipalities of Limassol district

Origin (collection year)	Migrants from:			
	Limassol (2022)	Mesa Geitonia (2022)	Germasogeia (2022)	Limassol (2023)
Limassol (2022)	0.76	0.12	0.07	0.06
Mesa Geitonia (2022)	0.10	0.76	0.07	0.07
Germasogeia (2022)	0.08	0.09	0.78	0.06
Limassol (2023)	0.11	0.11	0.08	0.71

The dynamics of genotypes among the three municipalities continued during the seasonal transition from October 2022 to April 2023. Limassol appears to have received genotypes from 2022 (M = 0.11) as well as from Mesa Geitonia (M = 0.11) and, to a lesser extent, from Germasogeia (M = 0.08).

### Degree of ancestry between Cypriot mosquitoes and Central Mediterranean populations

**Fig. 3:**
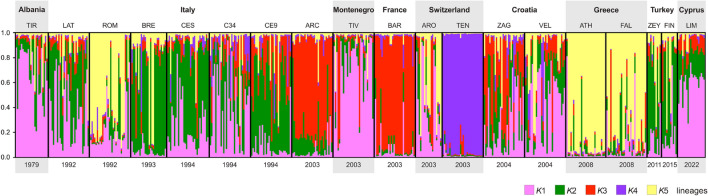
Representation of the coancestry of *Aedes albopictus* mosquitoes from Cyprus and those from the other Central Mediterranean populations included in the study. Dates of invasion in the different regions are shown in the lower part of the figure. See Table 5 for population code. * K*, Lineage/cluster

A comprehensive analysis of the SSR individual profiles of Cypriot mosquitoes from Limassol district (Limassol, Mesa Geitonia and Germasogeia combined) and those of Mediterranean mosquitoes from Italy, France, Switzerland, Albania, Montenegro, Croatia, Greece and Turkey indicates that five lineages/clusters (*K*1–*K*5) represent the most parsimonious partitioning of ancestry among the genomes of these mosquitoes (Table 5; Fig. 3). Many Mediterranean populations appear to be fragmented among the lineages, such as the North and Central Italian populations (Cesena and Rome, respectively) and the Balkan populations (Velika Gorica and Zagreb in Croatia).

**Table 5:** Average coefficient of ancestry estimated for the Cypriot mosquitoes and the Central Mediterranean populations

Country	Population	Code	Lineages/clusters					*N*
			*K*1	*K*2	*K*3	*K*4	*K*5	
Albania	Tirana	TIR	0.71	0.15	0.05	0.01	0.08	24
Italy	Brescia	BRE	0.05	0.63	0.07	0.04	0.20	26
	Cesena	CES	0.28	0.54	0.07	0.04	0.07	31
	Cesena3 + 4	C34	0.40	0.30	0.14	0.09	0.07	30
	Cesena9	CE9	0.09	0.53	0.25	0.06	0.08	30
	Latina	LAT	0.14	0.20	0.04	0.02	0.60	30
	Rome	ROM	0.38	0.38	0.11	0.02	0.10	30
	Arco	ARC	0.04	0.17	0.72	0.02	0.04	30
Montenegro	Tivat	TIV	0.69	0.14	0.11	0.01	0.05	30
Switzerland	Tenero Contra	TEN	0.07	0.05	0.82	0.04	0.02	30
	Arogno	ARO	0.35	0.16	0.14	0.02	0.34	19
France	Bar sur Loup	BAR	0.01	0.01	0.02	0.94	0.01	30
Croatia	Velika Gorika	VEL	0.25	0.22	0.38	0.06	0.08	30
	Zagreb	ZAG	0.46	0.19	0.13	0.07	0.16	30
Greece	Faliro	FAL	0.05	0.06	0.02	0.01	0.87	29
	Athens	ATH	0.09	0.09	0.07	0.02	0.73	30
Turkey	Zeytinburnu	ZEY	0.15	0.54	0.06	0.02	0.23	10
	Findikli	FIN	0.19	0.51	0.07	0.02	0.21	11
Cyprus	Limassol district	LIM	0.69	0.15	0.11	0.02	0.04	20

Average coefficient of ancestry was obtained using *K* = 5 in STRUCTURE

The Cypriot mosquitoes from Limassol district are also fragmented but they have the highest ancestry in lineage *K*1 (0.69), shared with the Balkan mosquitoes from Albania (Tirana 0.71), Montenegro (Tivat 0.69) and Croatia (Zagreb 0.46), as well as the Italian populations from Cesena3 + 4 (0.40) and Rome (0.38). It is of interest to note that the mosquitoes from Limassol district do not share ancestry with Greek and Turkish neighbouring populations (Table 5).

### Inferred migration rates from Mediterranean areas into Cyprus

Assuming possible recent movements of mosquitoes between Central Mediterranean countries and Cyprus, Bayesian data suggest a recent, unidirectional migration of mosquitoes from the Balkans (Montenegro [Tivat]), into the Limassol district, with an M value of 0.18 (Table 6).

**Table 6:** Migration rates of *Aedes albopictus* mosquitoes estimated between the Central Mediterranean populations and those of Limassol district

Code for mosquito population^a^	Albania	Italy							Montenegro	Switzerland		France	Croatia		Greece		Turkey		Cyprus
	TIR	BRE	CES	C34	CE9	ROM	LAT	ARC	TIV	BAR	ARO	TEN	ZAG	VEL	ATH	FAL	ZEY	FIN	LIM
TIR	0.67	0.01	0.01	0.01	0.01	0.01	0.02	0.01	0.18	0.01	0.01	0.01	0.01	0.01	0.01	0.01	0.01	0.01	0.01
BRE	0.01	0.67	0.01	0.01	0.01	0.01	0.19	0.01	0.01	0.01	0.01	0.01	0.01	0.01	0.01	0.01	0.01	0.01	0.01
CES	0.01	0.01	0.67	0.01	0.01	0.01	0.20	0.01	0.01	0.01	0.01	0.01	0.01	0.01	0.01	0.01	0.01	0.01	0.01
C34	0.01	0.01	0.01	0.67	0.01	0.01	0.12	0.01	0.10	0.01	0.01	0.01	0.01	0.01	0.01	0.01	0.01	0.01	0.01
CE9	0.01	0.01	0.01	0.01	0.67	0.01	0.21	0.01	0.01	0.01	0.01	0.01	0.01	0.01	0.01	0.01	0.01	0.01	0.01
ROM	0.01	0.01	0.01	0.01	0.01	0.67	0.01	0.01	0.20	0.01	0.01	0.01	0.01	0.01	0.01	0.01	0.01	0.01	0.01
LAT	0.01	0.01	0.01	0.01	0.01	0.01	0.86	0.01	0.02	0.01	0.01	0.01	0.01	0.01	0.01	0.01	0.01	0.01	0.01
ARC	0.01	0.01	0.01	0.01	0.01	0.01	0.02	0.86	0.01	0.01	0.01	0.01	0.01	0.01	0.01	0.01	0.01	0.01	0.01
TIV	0.01	0.01	0.01	0.01	0.01	0.01	0.01	0.01	0.87	0.01	0.01	0.01	0.01	0.01	0.01	0.01	0.01	0.01	0.01
BAR	0.01	0.01	0.01	0.01	0.01	0.01	0.02	0.20	0.01	0.67	0.01	0.01	0.01	0.01	0.01	0.01	0.01	0.01	0.01
ARO	0.01	0.01	0.01	0.01	0.01	0.01	0.01	0.02	0.16	0.01	0.68	0.01	0.01	0.01	0.01	0.01	0.01	0.01	0.01
TEN	0.01	0.01	0.01	0.01	0.01	0.01	0.01	0.01	0.01	0.01	0.01	0.87	0.01	0.01	0.01	0.01	0.01	0.01	0.01
ZAG	0.01	0.01	0.01	0.01	0.01	0.01	0.20	0.01	0.01	0.01	0.01	0.01	0.67	0.01	0.01	0.01	0.01	0.01	0.01
VEL	0.01	0.01	0.01	0.01	0.01	0.01	0.03	0.01	0.18	0.01	0.01	0.01	0.01	0.67	0.01	0.01	0.01	0.01	0.01
ATH	0.01	0.01	0.01	0.01	0.01	0.01	0.01	0.01	0.02	0.01	0.01	0.01	0.01	0.01	0.86	0.01	0.01	0.01	0.01
FAL	0.01	0.01	0.01	0.01	0.01	0.01	0.01	0.01	0.02	0.01	0.01	0.01	0.01	0.01	0.20	0.67	0.01	0.01	0.01
ZEY	0.01	0.01	0.01	0.01	0.01	0.01	0.08	0.01	0.05	0.01	0.01	0.01	0.01	0.01	0.02	0.01	0.68	0.01	0.01
FIN	0.01	0.01	0.01	0.01	0.01	0.01	0.08	0.01	0.05	0.01	0.01	0.01	0.01	0.01	0.03	0.01	0.01	0.68	0.01
LIM	0.01	0.01	0.01	0.01	0.01	0.01	0.01	0.01	0.18	0.01	0.01	0.01	0.01	0.01	0.01	0.01	0.01	0.01	0.68

^a^See Table 5 for definition of codes used for each mosquito population

## Discussion

Several key findings emerged from applying a genetic and demographic approach to a sample of 20 of the 69 *Ae. albopictus* mosquitoes recovered in three municipalities of the Limassol district between October and November 2022 and in April 2023.

Firstly, there was a high degree of individual variability and evidence of differentiation among the mosquito populations from the different municipalities. Secondly, a general pattern of mosquito movements within the Limassol district was observed. Thirdly, there was a notable degree of ancestry between the Cypriot mosquito population and those of certain Balkan countries. It was possible to identify the potential source area of *Ae. albopictus* incursion into Cyprus, namely Limassol port.

### Looking inside Cyprus: genetic status and signs of differentiation among municipalities

**Fig. 4:**
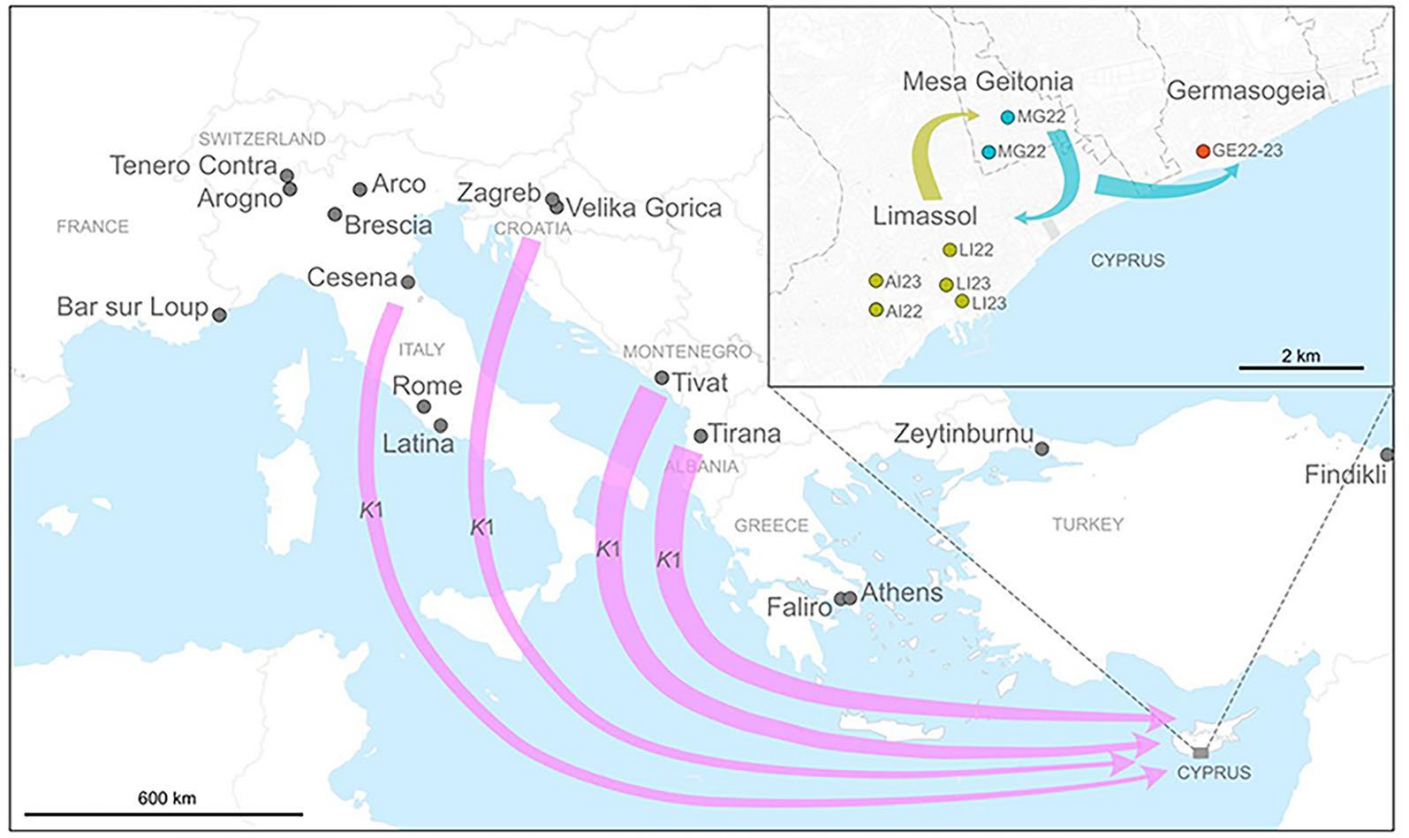
Geographical representation of migration rates among municipalities within the Limassol district and across the Mediterranean Sea. The only population identified as a donor to the *Ae. albopictus* population of Limassol, Cyprus, originates from Tivat, Montenegro

After several complaints that also included photographs of specimens taken during September 2022, on 3 October 2022 six male *Ae. albopictus* mosquitoes were captured in BG traps in Limassol and 27 male and 16 female *Ae. albopictus* mosquitoes were captured in BG traps in Mesa Geitonia. This high number of captured adult mosquitoes suggested the presence of *Ae. albopictus* populations established in these municipalities [38]. The genetic status of these mosquitoes supports such a hypothesis. There are no signs of genetic drift due to recent founder effects in these mosquitoes, which appear to be highly polymorphic, with 78% of molecular variance present within individuals and relatively high estimates of effective alleles (*N*_e_) and expected heterozygosity (*H*_e_) within the municipalities. Moreover, Bayesian analyses of recent migration rates showed ongoing mosquito dispersal among the municipalities (Fig. 4). If this gene flow contributed towards buffering the erosion of variability due to eventual past founder effects, this dispersal confirms the presence of established populations within the Limassol district. The mosquito movement continued and increased over time, as evidenced for the Limassol municipality between October 2022 and April 2023, contributing to an increase in variability (*N*_e_ from 1.8 to 2.2) and, consequently, the adaptive potential for the stabilisation of the population and subsequent overland dispersal. The potential adaptability of this mosquito species and the fact that Cyprus hosts a diverse range of habitats would allow it to expand its dispersal areas [40, 41].

The sites where these mosquitoes were collected are near the marina and port of Limassol, as well as near the busy road network of Mesa Geitonia, which is very close to Limassol City, and in Germasogeia, located in the eastern part of the district. It has been suggested that the introduction of this mosquito in the Limassol area occurred through trade and maritime transport to the Limassol port, with possible subsequent dispersal by vehicles [28, 38].

The mosquitoes from Limassol and neighbouring Mesa Geitonia appear to be highly genetically related, as shown in the PCoA analysis (Fig. 2), with fairly high reciprocal ongoing movements, but they display a very slight differentiation versus the mosquitoes from Germasogeia. Moreover, the movements towards this last municipality are reduced. Whether the differentiation of Germasogeia mosquitoes is consequent to subsequent dispersal from the proposed entry point is an open question. Considering that Germasogeia is a highly international tourist area, we cannot exclude the possibility of several independent introduction events.

### Demographic history of Cypriot mosquitoes

The presence of *Ae. albopictus* in the Mediterranean region is the result of a complex invasion pattern, with most established populations being admixtures resulting from independent introductions of unrelated genomes scattered across time [1–3]. This has generated genetic heterogeneity among populations, such that neighbouring populations may display different degrees of ancestry across lineages or sublineages.

As illustrated in Fig. 3, this heterogeneity is evident also in the Central Mediterranean area, where the mosquito genomes from Italy and the Balkans are fragmented among the five detected lineages. The ancestry profiles of the adventive Cypriot mosquitoes suggest that their genetic backgrounds are also a mixture of Mediterranean genotypes. They display high genetic connectivity and ancestry with populations from the Balkans and Italy. It is worth noting that Mediterranean countries such as Albania, Montenegro, Croatia and Italy are important trading partners of Cyprus. Indeed, Cesena, which shares the *K*1 designation with the Limassol district, is an Italian city neighbouring the port of Ravenna, a leading hub for trade with Central and Eastern Mediterranean locations. Limassol, with its two ports, serves as the primary centre for trading activities in Cyprus [77] and is also an important terminal for tourist cruises [78].

The trade connections between the Limassol ports and countries where *Ae. albopictus* is already established, such as those we considered here, and tourist activity are important factors to consider when evaluating the possible pathways for the entry of *Ae. albopictus* into Cyprus [40]. Moreover, a previous study demonstrated that international maritime and shipping networks have been instrumental in facilitating the introductions of *Ae. albopictus* and *Ae. aegypti* into the USA Gulf Coast [79].

The suggestion that the port of Limassol may have been the entry point for the incursion of *Ae. albopictus* into Cyprus is also supported by our Bayesian analyses of recent migration. These analyses indicate a unidirectional movement of genotypes towards the Limassol municipality (including the port) from the Balkan region, specifically Tivat, Montenegro (Fig. 4). Intensive cargo shipping activities, including rubber trading, are ongoing between Montenegro and Cyprus [80]. Additionally, Tivat serves as a hub for nautical tourism in the Southern Adriatic Sea.

### Incursion of *Ae. albopictus* into Cyprus: potential risks for arbovirus outbreaks

Given the chaotic global dispersion of *Ae. albopictus*, both population ancestry and admixture may contribute to creating conditions for the efficient and differential transmission of arboviruses, potentially leading to outbreaks [2]. Vega Rua and colleagues [3] demonstrated that the demographic history of *Ae. albopictus* populations influences their competence for CHIKV. Specifically, the history of adventive populations appears to be associated with CHIKV genotypes in a genotype-by-genotype interaction that affects their vector competence.

In this context, the genetic relationships linking the Cypriot mosquitoes to Mediterranean populations must be considered (Fig. 4). Indeed, Mariconti and colleagues [21] showed that the Central Mediterranean populations considered in the present study were susceptible to CHIKV (ECSA lineage), DENV-1 and ZIKV, but had the highest vector competence for CHIKV (infection and transmission). There are interpopulation variations, such as those seen in Cesena in Italy, Tivat in Montenegro, Velika Gorica and Zagreb in Croatia and Faliro in Greece. For DENV, the populations showed almost similar competence profiles with moderate levels of infection and transmission, with exceptions being Cesena, Faliro and Croatia, which showed slightly higher levels. On the other hand, all populations had low competence for ZIKA.

Considering that Cypriot mosquitoes are a mixture of these Mediterranean genomes and that the degree of competence for different arboviruses depends on specific combinations between vector and pathogen genotypes, as well as temperature and environmental factors [4, 81, 82], it is important to recognise that the presence of this mosquito in Cyprus may create conditions for the risk of outbreaks. Indeed, as Vasquez and colleagues [38] mentioned, the human population in Cyprus is immunologically naive to arboviruses transmitted by *Aedes* mosquitoes such as *Ae. albopictus*. Moreover, the island is a popular tourist destination throughout the year.

## Conclusions

Several considerations emerge from a comprehensive analysis of our data. Firstly, we acknowledge that our study is based on a small sample of 20 adventive specimens. Moreover, these specimens were collected in a delimited temporal window, between October 2022 and April 2023. This time frame may not fully capture the seasonal genetic variability, population dynamics and dispersal patterns of *Ae. albopictus* in Cyprus based on other available data [55, 83]. Seasonal fluctuations in mosquito abundance and movement are influenced by temperature, precipitation and human activity, which can vary significantly across different times of the year [84–86].

However, our sample is part of a larger sample of 69 mosquitoes, and this larger sample represents the first recorded presence of *Ae. albopictus* in Cyprus. We are confident that our smaller sample is representative of the mosquito genomes collected near Limassol port, which is posited as the possible entry point, a hypothesis our data seem to confirm. Future studies incorporating year-round sampling would provide a more comprehensive understanding of the demographic and genetic structure of *Ae. albopictus* and its potential for establishment and adaptation across diverse habitats in Cyprus.

Moreover, the data presented here constitute the first demographic analysis of the *Ae. albopictus* incursion in Cyprus. This will be an important first step for future studies on the evolutionary processes of adaptation that *Ae. albopictus* may undergo during its potential expansion within Cyprus.

Regarding the risks of arbovirus outbreaks due to the presence of *Ae. albopictus* in Cyprus, understanding the demographic history, which may reflect the viral competence [3, 21] of these initial adventive mosquitoes, will be crucial for predicting and preventing potential human health risks.

## Supplementary Information

**Additional file 1: Table S1. Genetic variability of the** ***Aedes albopictus*** **detected in the municipalities of Limassol district.**.

## Data Availability

Individual genotype data are available on Open Science Framework (OSF) (https://osf.io/gpuwk/?view_only = 6bf91ff58be1421a8c216cb6ae65cc0a).
