# Supplementary material for: On the tracks of an uninvited guest, the Asian tiger mosquito, Aedes albopictus in Cyprus
Source: Parasit Vectors. 2025 Feb 4;18:39. doi: 10.1186/s13071-024-06651-5 (PMC11796127; doi:10.1186/s13071-024-06651-5)
Supplement: Supplementary file 1 — Additional file 1: Table S1. Genetic variability of the Aedes albopictus detected in the municipalities of Limassol district.. [file 13071_2024_6651_MOESM1_ESM.docx]

**Table S1**. Genetic variability of the *Ae. albopictus* detected in the municipalities of Limassol district.

| **Municipality** | **Collection date** | ***N*_a_** | ***N*_a_/*N*** | ***N*_e_** | ***H*_o_** | ***H*_e_** | ***F*** |
| --- | --- | --- | --- | --- | --- | --- | --- |
| Limassol | 2022 | 2.18 | 0.45 | 1.82 | 0.36 | 0.36 | -0.02 |
| Mesa Geitonia | 2022 | 2.00 | 0.42 | 1.74 | 0.36 | 0.35 | -0.08 |
| Germasogeia | 2022 | 1.64 | 0.55 | 1.49 | 0.15 | 0.24 | 0.24 |
| Limassol | 2023 | 2.73 | 0.46 | 2.22 | 0.38 | 0.41 | 0.08 |

*N*_a_, number of alleles; *N*_a_/*N*, number of alleles per individual; *N*_e_, number of effective alleles;*H*_o_, observed heterozygosity; *H*_e_, expected heterozygosity; *F*, fixation index
